# Supplementary material for: High resolution clonal architecture of hypomutated Wilms tumours
Source: Nat Commun. 2025 May 29;16:4647. doi: 10.1038/s41467-025-59854-4 (PMC12122882; doi:10.1038/s41467-025-59854-4)
Supplement: Supplementary file 1 — Supplementary Information [file 41467_2025_59854_MOESM1_ESM.pdf]

# Supplementary Information

Contents

- Supplementary Figures 1-10……………………………………………………………………..1
- Supplementary Note 1. Morphological description of microscopy of Wilms tumours…………..12
- Supplementary Note 2. Description of the role of morphology in our case selection……………12
- Supplementary Note 3. Laser capture microdissection of the tumours…………………………..13
- Supplementary Note 4. Comparison of mutation burden with morphology…………………..…13
- Supplementary Note 5. Immunohistochemistry of the *FOXR2*-mutant tumours……………..…14
- Supplementary Note 6. Overall mutation burden within the tumour……………………..………16
- Supplementary References……………………………………..…………………...………....19

## SUPPLEMENTARY FIGURES

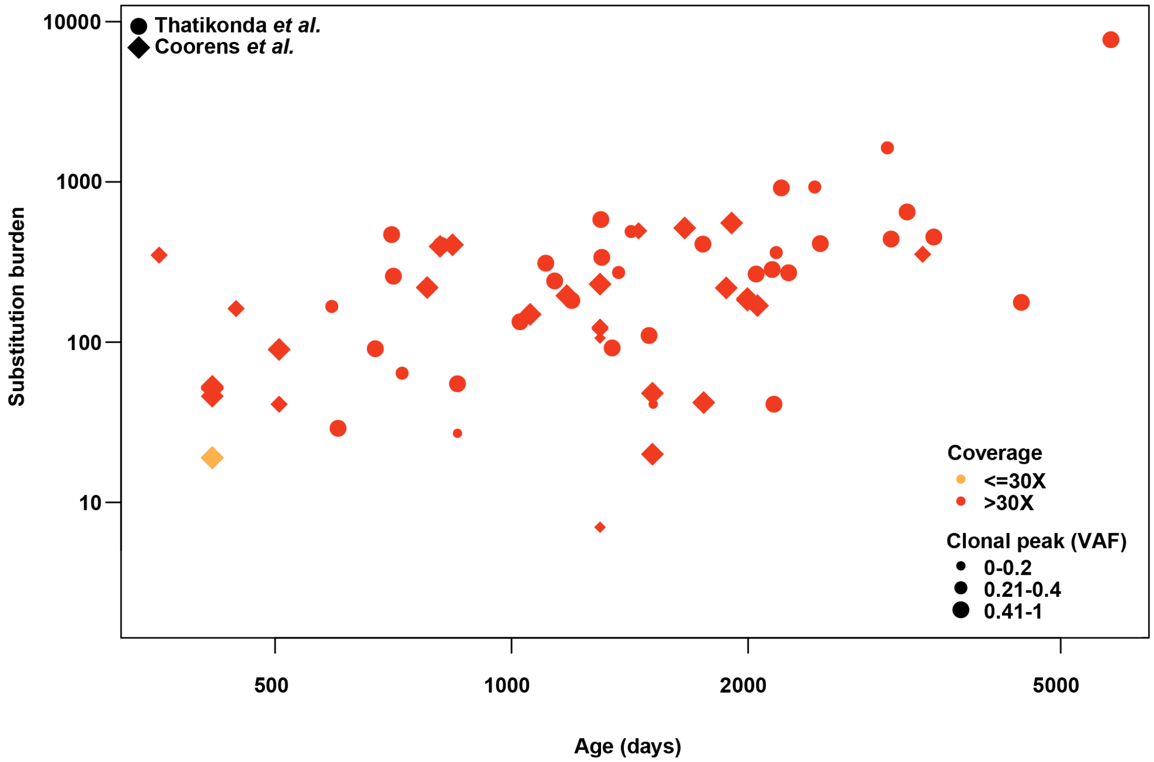

**Supplementary Fig. 1. Substitution burden of Wilms tumours accounting for age, tumour purity, and coverage.** Substitution burden from whole genome sequencing of two previously published cohorts of Wilms tumours[6,11] plotted against patient age, with point size and colour determined by coverage and tumour purity (which is twice the VAF) respectively. Source data are provided as a Source Data file.

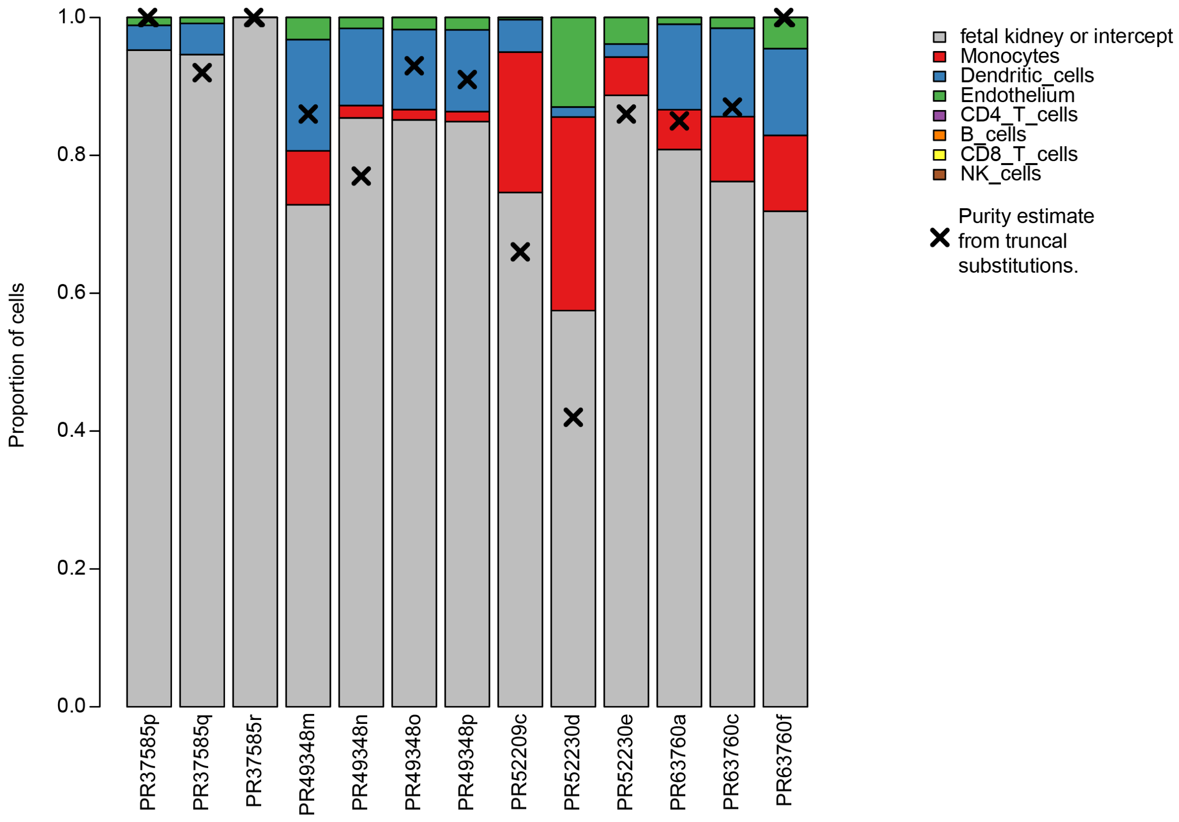


**Supplementary Fig. 2. Cell signal mapping of the transcriptomes of Wilms tumours.** Bulk RNA sequencing of Wilms tumour samples mapped onto a kidney single cell reference[40] (Methods) estimates the proportion of each biopsy made up of different cell types. Mapping onto fetal kidney or not assigned to any cell type (the intercept) is likely to represent tumour cells or adjacent normal kidney. This analysis shows that there is not a significant contribution from cells that have high mutation rates (such as B cells) that could be skewing the nanoseq mutation burden. A point estimate of the tumour purity based on the allele fraction of truncal tumour substitutions in each biopsy is overlaid. Source data are provided as a Source Data file.

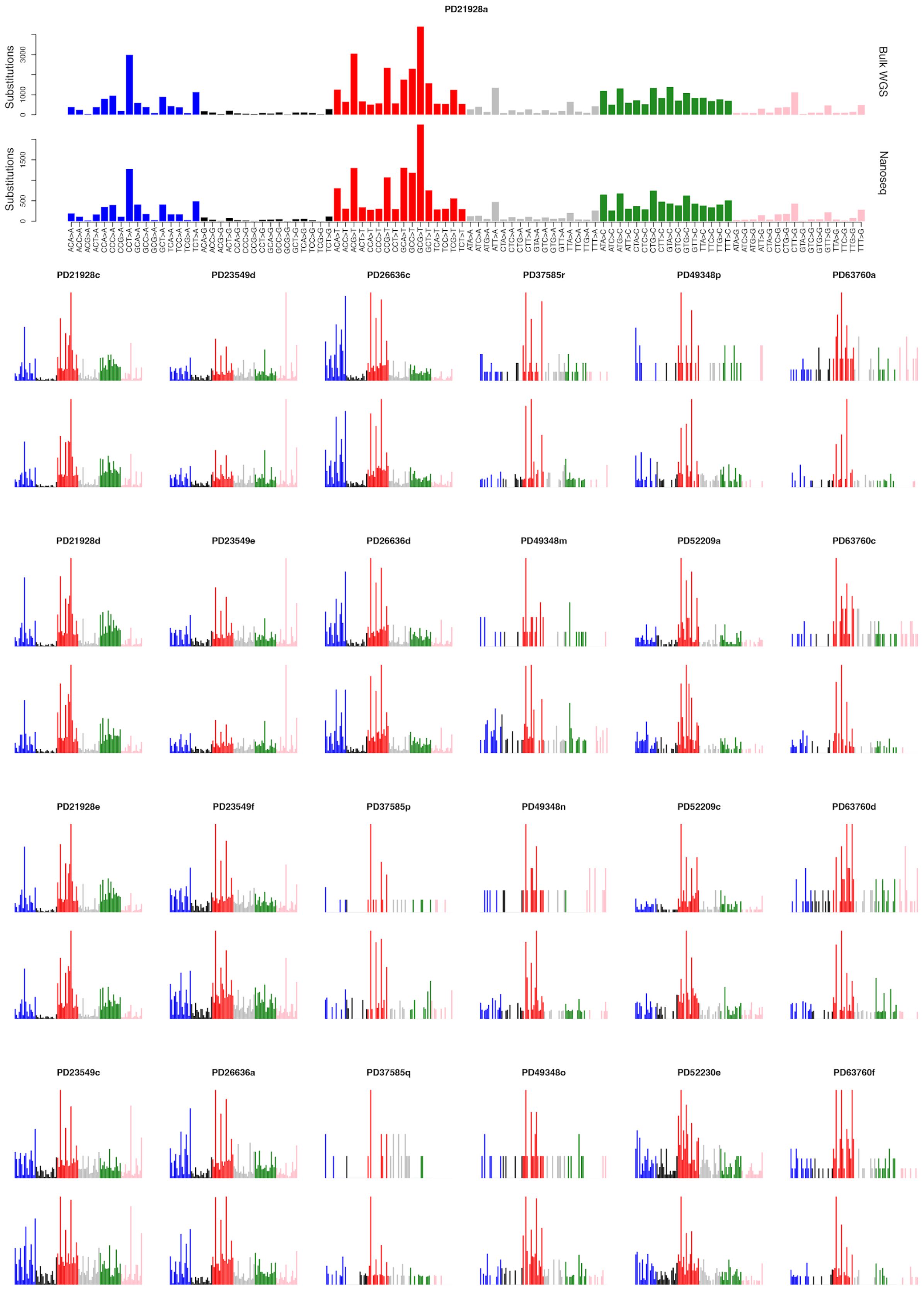
PD21928a
Substitutions
0
1000
3000
Bulk WGS
Substitutions
0
500
1500
Nanoseq
PD21928c
PD23549d
PD26636c
PD37585r
PD49348p
PD63760a
PD21928d
PD23549e
PD26636d
PD49348m
PD52209a
PD63760c
PD21928e
PD23549f
PD37585p
PD49348n
PD52209c
PD63760d
PD23549c
PD26636a
PD37585q
PD49348o
PD52230e
PD63760f

**Supplementary Fig. 3. Trinucleotide spectra of tumour samples.** For each tumour sample for which both whole genome bulk sequencing and nanoseq calls were available, the 96-context trinucleotide spectra of substitutions are plotted. One sample is show at the top with axes as an example. All subsequent samples are shown in the same format, with bulk whole genome sequencing spectra above nanoseq spectra. The spectra are similar, suggesting that the nanoseq estimates are not raised by contamination with highly mutated non-tumour cells, which would be likely to have different spectra. WGS, whole genome sequencing. Source data are provided as a Source Data file.

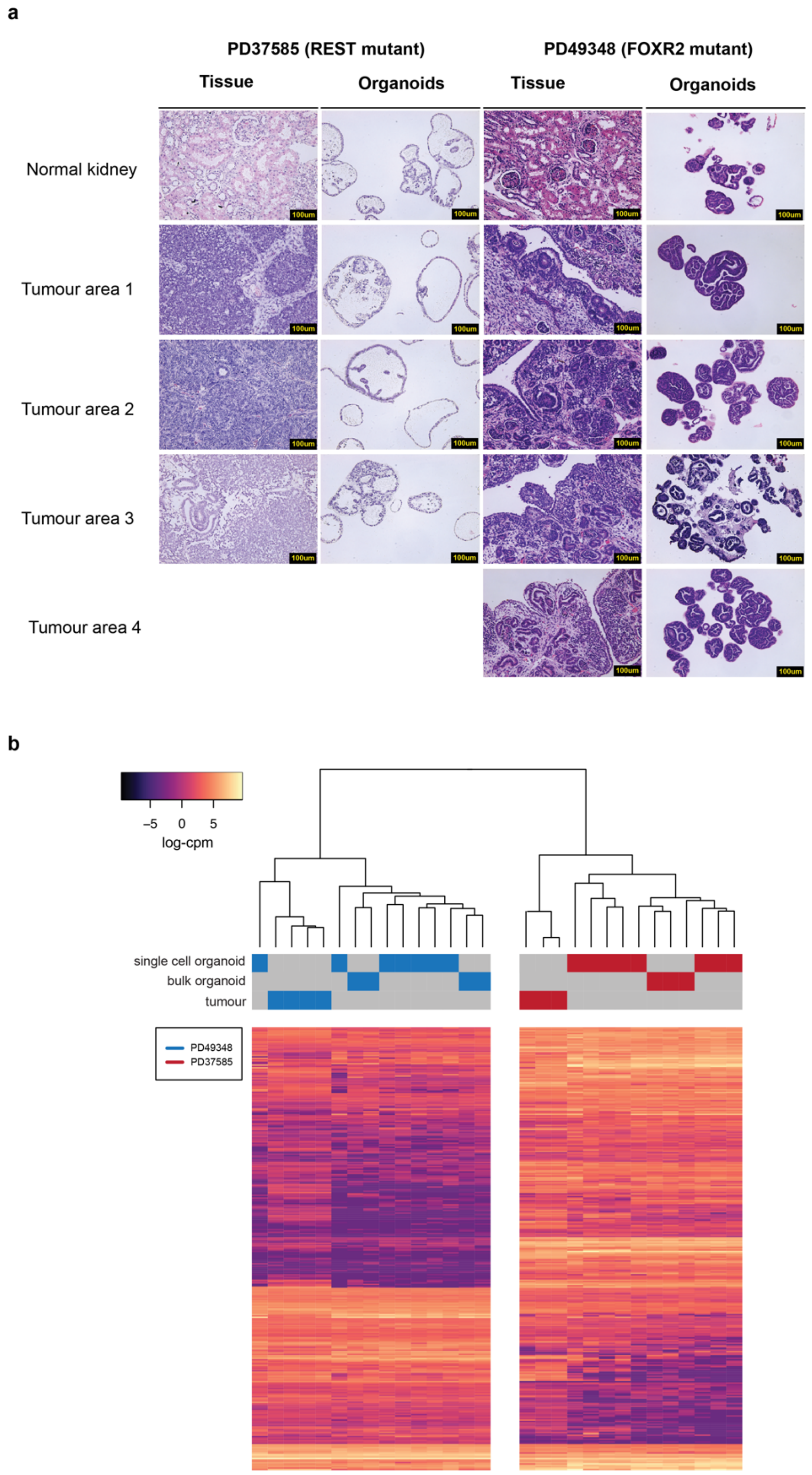


**Supplementary Fig. 4.** Single-cell-derived organoids from infant Wilms tumours. **a**, the morphology of organoids is shown next to the tumour areas from which they were derived. **b**, hierarchical clustering of RNAseq data of bulk tumours, polyclonal organoids, and single-cell-derived organoids. The single-cell-derived and polyclonal organoids intermingle, suggesting that the single cell cloning step does not significantly skew the organoid population. Source data are provided as a Source Data file.

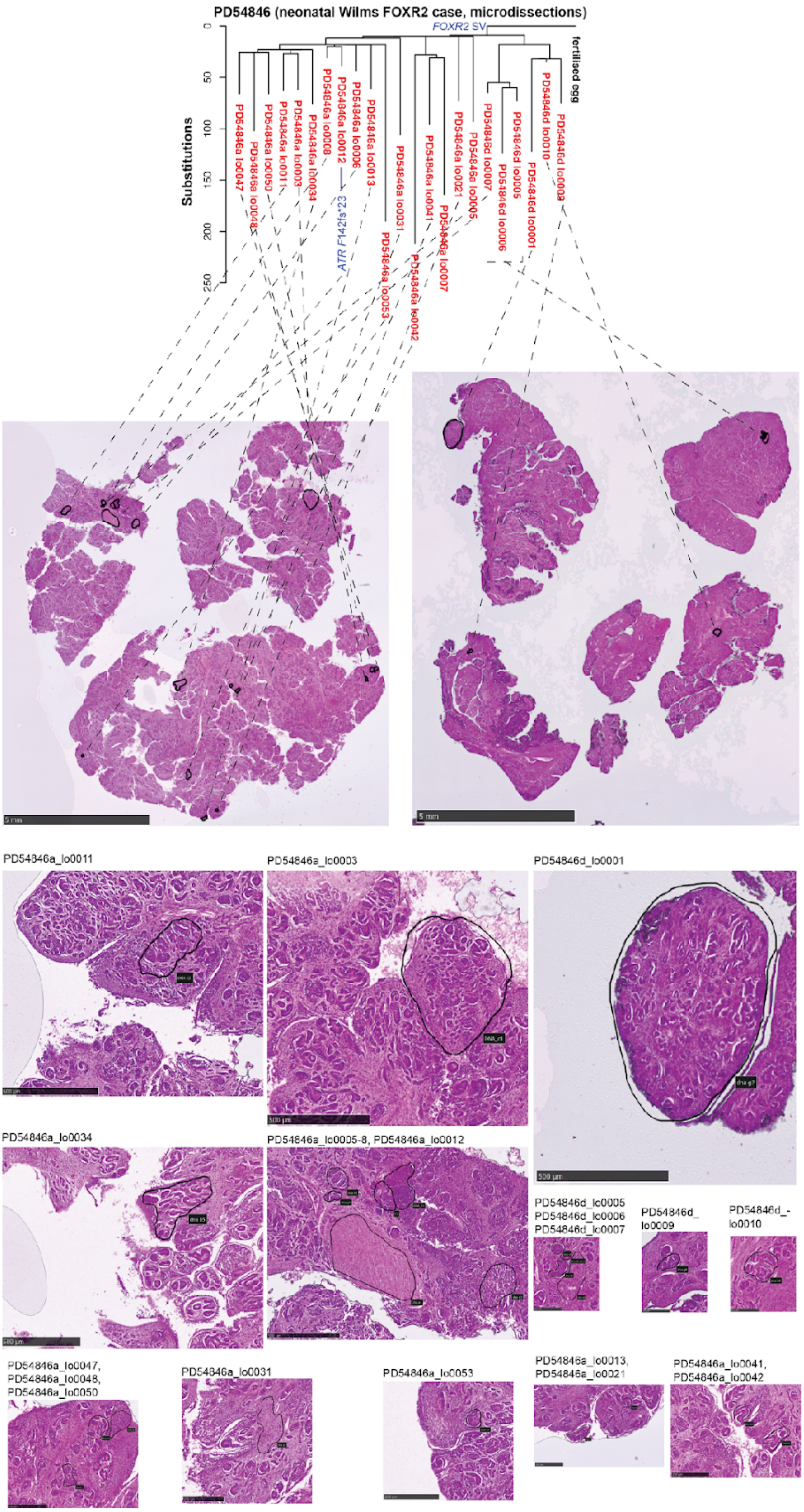
PD54846 (neonatal Wilms FOXR2 case, microdissections)
FOXR2 SV
fertilised egg
Substitutions
0
50
100
150
200
250
PD54846a lo0047
PD54846a lo0048
PD54846a lo0050
PD54846a lo0011
PD54846a lo0003
PD54846a lo0034
PD54846a lo0008
PD54846a lo0012
PD54846a lo0006
PD54846a lo0013
ATR F142fs*23
PD54846a lo0031
PD54846a lo0053
PD54846a lo0041
PD54846a lo0042
PD54846a lo0021
PD54846a lo0007
PD54846a lo0005
PD54846d lo0007
PD54846d lo0005
PD54846d lo0006
PD54846d lo0001
PD54846d lo0010
PD54846d lo0009
PD54846a_lo0011
PD54846a_lo0003
PD54846d_lo0001
PD54846a_lo0034
PD54846a_lo0005-8, PD54846a_lo0012
PD54846d_lo0005
PD54846d_lo0006
PD54846d_lo0007
PD54846d_ lo0009
PD54846d_- lo0010
PD54846a_lo0047,
PD54846a_lo0048,
PD54846a_lo0050
PD54846a_lo0031
PD54846a_lo0053
PD54846a_lo0013,
PD54846a_lo0021
PD54846a_lo0041,
PD54846a_lo0042

**Supplementary Fig. 5.** Laser capture microdissection of PD54846, a *FOXR2*-rearranged infant Wilms tumour. This experiment was carried out once. **a**, the phylogeny derived from whole genome sequencing of laser capture microdissections is shown above a low power image of the tissues before microdissection, with each phylogeny tip matched to the structure that was sequenced. **b**, higher magnification images of each microdissected area are shown. Source data are provided as a Source Data file.

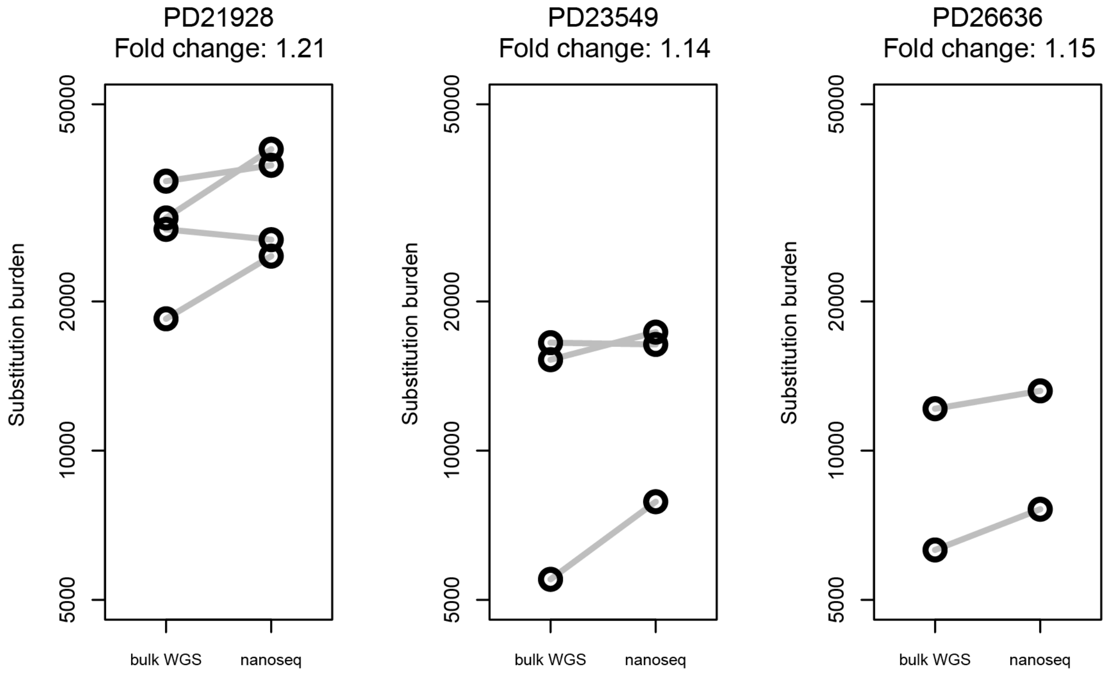


**Supplementary Fig. 6. Comparison of bulk whole genome sequencing and nanoseq substitution burdens for colorectal cancers.** For three previously published[12] colorectal cancers, nanoseq data were newly generated and the substitution burden compared. The bulk estimate has been corrected to 30X coverage (Methods). Please note that there is no adjustment for copy number. Each point represents one biopsy from a tumour, and where the same biopsy has been sequenced using both methodologies, points are linked by grey lines. The fold change between the median burden from nanoseq and bulk whole genome sequencing is shown above. WGS, whole genome sequencing. Source data are provided as a Source Data file.

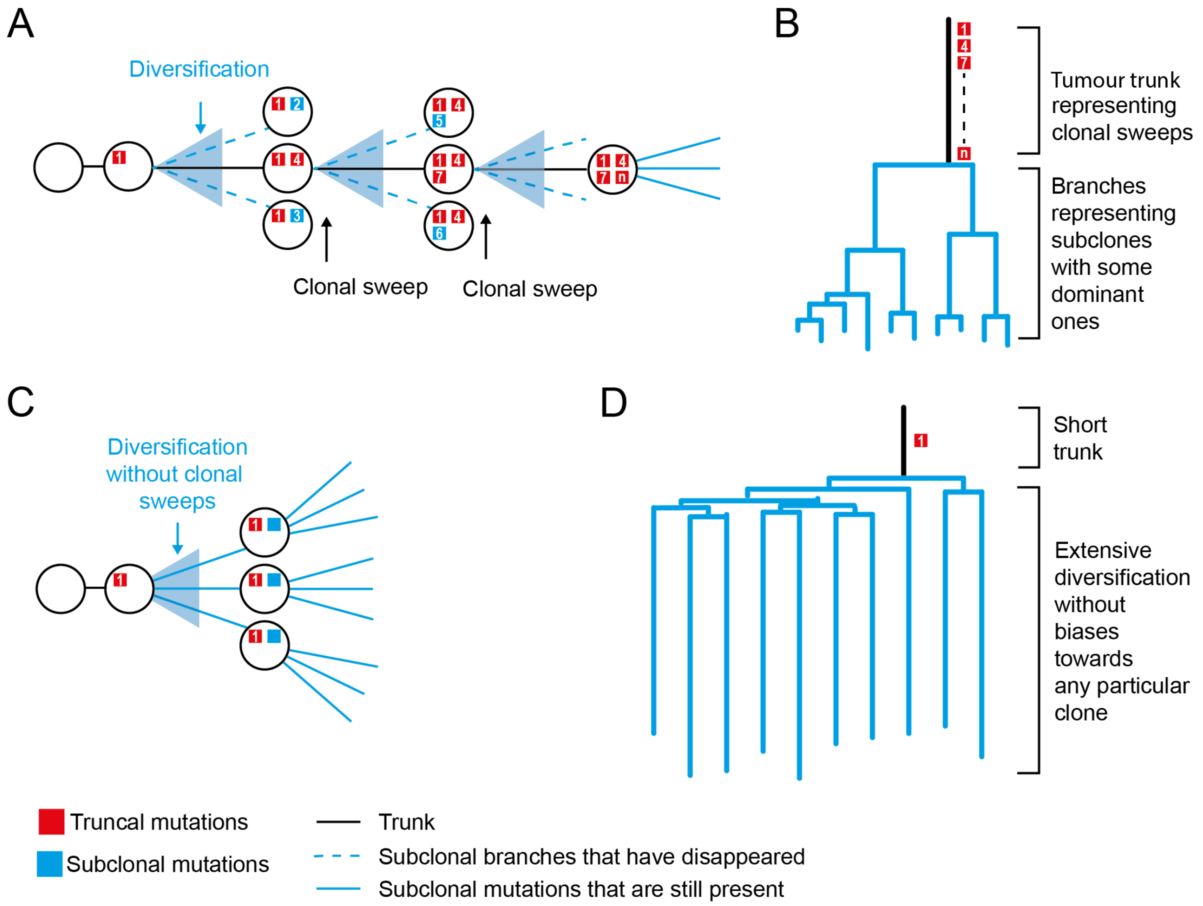


**Supplementary Fig. 7. Schematics of clonal diversification patterns and their resultant phylogenies.** Sequential waves of clonal diversification and sweeps with ongoing competition between subclones (**a**) results in a long phylogenetic trunk with large ramifications (**b**). This matches what we observe in the schoole age Wilms tumours and the adult colorectal cancers. In contrast, early clonal diversification and subsequent parallel co-existence of subclones with little subclonal competition (**c**) results in a short trunk that rapidly splits into many long terminal branches (**d**). This matches what we observe in infant Wilms cases.

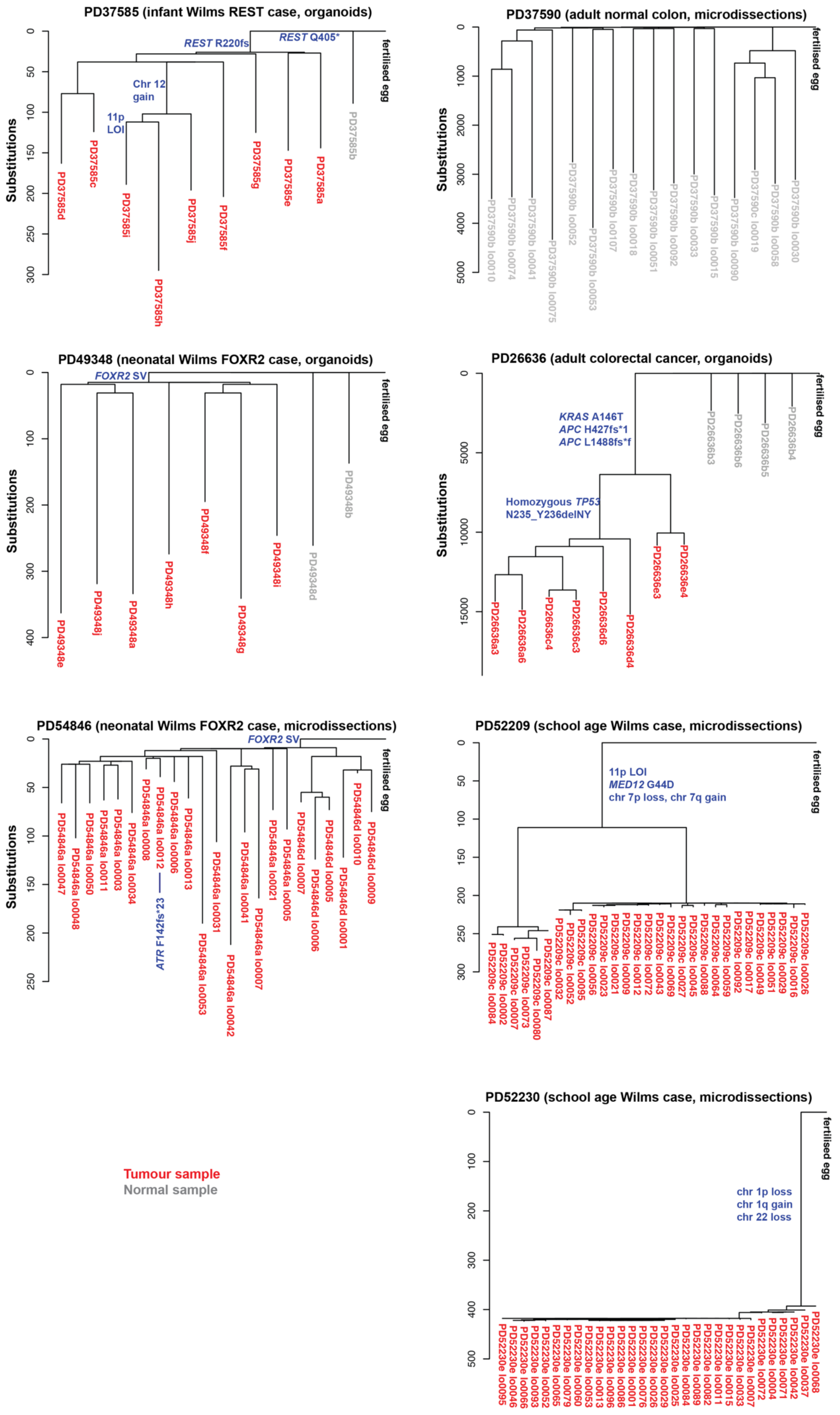
PD37585 (infant Wilms REST case, organoids)
Substitutions
REST R220fs
REST Q405*
Chr 12 gain
11p LOI
fertilised egg
PD37585d
PD37585c
PD37585i
PD37585h
PD37585j
PD37585f
PD37585g
PD37585e
PD37585a
PD37585b
PD37590 (adult normal colon, microdissections)
Substitutions
fertilised egg
PD37590b lo0010
PD37590b lo0074
PD37590b lo0041
PD37590b lo0075
PD37590b lo0052
PD37590b lo0053
PD37590b lo0107
PD37590b lo0018
PD37590b lo0051
PD37590b lo0092
PD37590b lo0033
PD37590b lo0015
PD37590b lo0090
PD37590c lo0019
PD37590b lo0058
PD37590b lo0030
PD49348 (neonatal Wilms FOXR2 case, organoids)
Substitutions
FOXR2 SV
fertilised egg
PD49348e
PD49348j
PD49348a
PD49348h
PD49348f
PD49348g
PD49348i
PD49348d
PD49348b
PD26636 (adult colorectal cancer, organoids)
Substitutions
KRAS A146T
APC H427fs*1
APC L1488fs*f
Homozygous TP53
N235_Y236delNY
fertilised egg
PD26636a3
PD26636a6
PD26636c4
PD26636c3
PD26636d6
PD26636d4
PD26636e3
PD26636e4
PD26636b3
PD26636b6
PD26636b5
PD26636b4
PD54846 (neonatal Wilms FOXR2 case, microdissections)
Substitutions
FOXR2 SV
ATR F142fs*23
fertilised egg
PD54846a lo0047
PD54846a lo0048
PD54846a lo0050
PD54846a lo0011
PD54846a lo0003
PD54846a lo0034
PD54846a lo0008
PD54846a lo0012
PD54846a lo0006
PD54846a lo0013
PD54846a lo0053
PD54846a lo0031
PD54846a lo0042
PD54846a lo0041
PD54846a lo0007
PD54846a lo0021
PD54846a lo0005
PD54846d lo0007
PD54846d lo0006
PD54846d lo0005
PD54846d lo0001
PD54846d lo0010
PD54846d lo0009
PD52209 (school age Wilms case, microdissections)
11p LOI
MED12 G44D
chr 7p loss, chr 7q gain
fertilised egg
PD52209c lo0084
PD52209c lo0002
PD52209c lo0007
PD52209c lo0073
PD52209c lo0080
PD52209c lo0087
PD52209c lo0032
PD52209c lo0052
PD52209c lo0095
PD52209c lo0056
PD52209c lo0023
PD52209c lo0021
PD52209c lo0009
PD52209c lo0012
PD52209c lo0072
PD52209c lo0043
PD52209c lo0069
PD52209c lo0027
PD52209c lo0045
PD52209c lo0088
PD52209c lo0064
PD52209c lo0059
PD52209c lo0092
PD52209c lo0017
PD52209c lo0049
PD52209c lo0051
PD52209c lo0029
PD52209c lo0016
PD52209c lo0026
PD52230 (school age Wilms case, microdissections)
chr 1p loss
chr 1q gain
chr 22 loss
fertilised egg
PD52230e lo0095
PD52230e lo0046
PD52230e lo0066
PD52230e lo0093
PD52230e lo0052
PD52230e lo0065
PD52230e lo0079
PD52230e lo0060
PD52230e lo0053
PD52230e lo0013
PD52230e lo0096
PD52230e lo0086
PD52230e lo0001
PD52230e lo0076
PD52230e lo0026
PD52230e lo0029
PD52230e lo0025
PD52230e lo0084
PD52230e lo0089
PD52230e lo0082
PD52230e lo0011
PD52230e lo0015
PD52230e lo0003
PD52230e lo0007
PD52230e lo0072
PD52230e lo0004
PD52230e lo0071
PD52230e lo0042
PD52230e lo0037
PD52230e lo0068
Tumour sample
Normal sample

**Supplementary Fig. 8. Phylogenies annotated with driver mutations and sample name.** Phylogenies are shown as in Figure 2, but indicating which sample relates to each branch, and with driver mutations annotated (Methods). Source data are provided as a Source Data file.

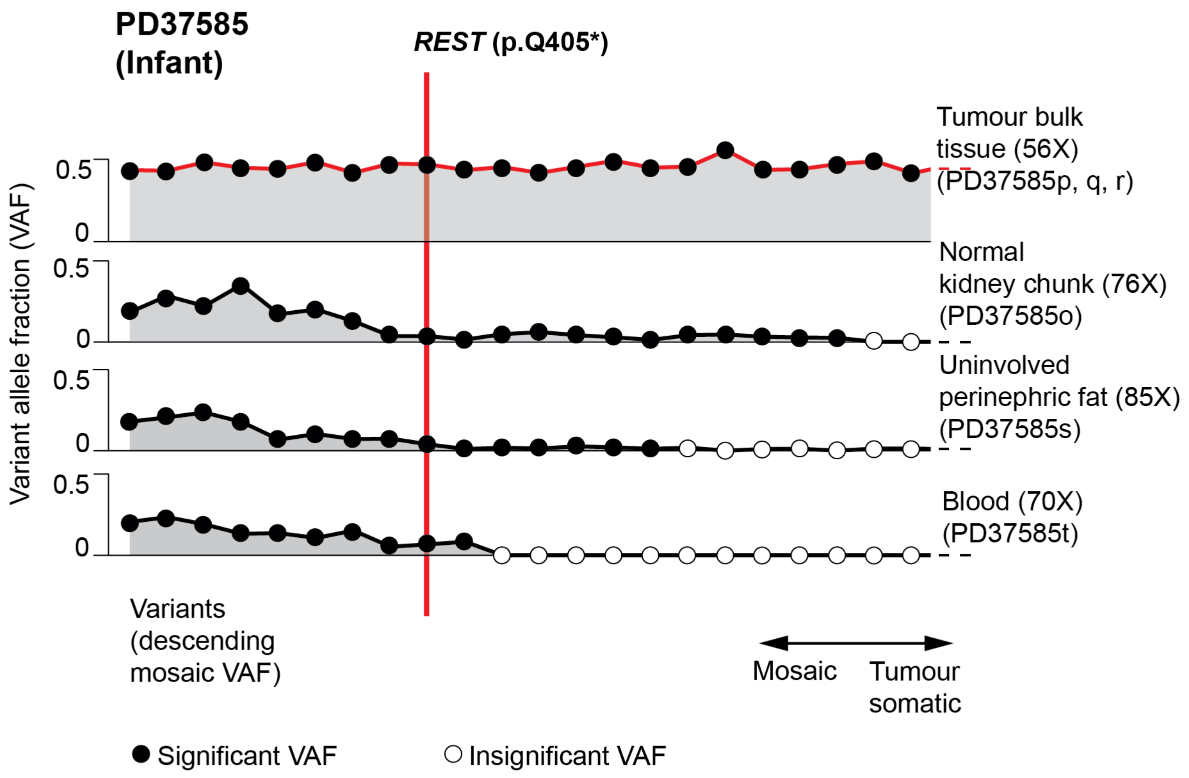


**Supplementary Fig. 9. A mosaic *REST* mutation in infant Wilms.** The allele fraction of somatic mutations from the Wilms tumour of a six-month-old was calculated in different normal tissues from the same patient. Mutations are plotted by decreasing combined allele fraction across normal tissues. Only the first 22 mutations are shown. Mutations that can be statistically distinguished from the error rate are coloured black, while those that cannot be distinguished from noise are coloured white. Mutations that are detectable in normal tissues, such as *REST* p.Q405* (a stop-gain mutation, indicated by a red vertical line), are embryonic mosaic mutations. Source data are provided as a Source Data file.

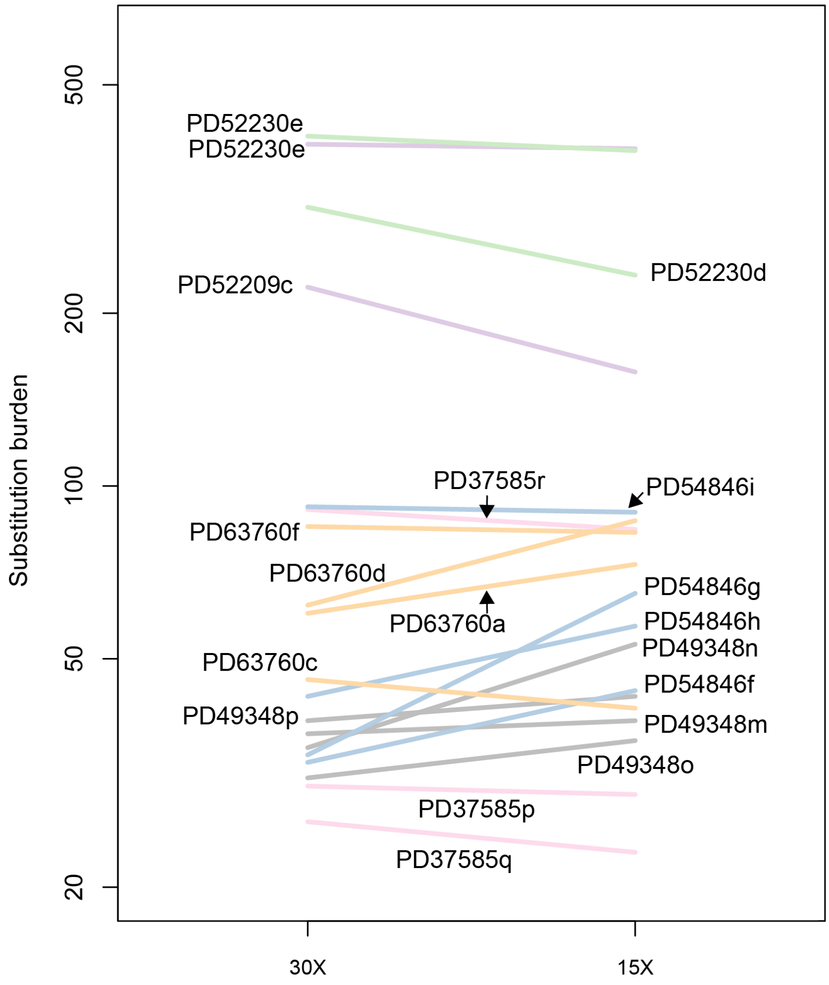


**Supplementary Fig. 10. Mutation burden from bulk whole genome sequencing with different levels of coverage.** For each tumour, the mutation burden is show with after downsampling to 30X and after downsampling to 15X (Methods), except for PD37585 samples that were sequenced at 17-20X and so could not be downsampled to 30X (non-downsampled data are shown for this patient). For some samples, surprisingly, the mutation burden increases with downsampling. This is likely a result of random sampling with small numbers of mutations. These data suggest that comparing PD37585 samples against 30X samples in Fig. 1 is reasonable. Samples are coloured by the patient from whom they derive. Source data are provided as a Source Data file.

**Morphological description of microscopy of Wilms tumours.**

Tumours from the infants PD49348, PD54846, and PD63760 all bore *FOXR2* rearrangements. The tumours of PD49348 and PD54846 had not been exposed to chemotherapy and are described together. The tumour of PD63760 had received neoadjuvant vincristine and actinomycin D, and is described separately.

Histopathological examination of PD49348 and PD54846 showed similar features. Both were well-circumscribed, encapsulated, intra-renal lesions with blastemal, epithelial and stromal elements (triphasic). The overall growth pattern in both lesions was described as fibroadenomatoid, with broad papillae covered by epithelium, and a more solid and nodular appearance where the tumors were continuous with background kidney. The epithelial elements in both cases showed prominent primitive glomeruloid and tubular structures. Several peri-lobar nephrogenic rests were identified within the background kidney in PD54846 (insufficient for the criteria of nephroblastomatosis). Both cases showed similar patterns of immunoreactivity within the tumor and background kidney, neither case showed anaplasia.

The tumour of PD63760 showed growth of broad fronds and nodules, many of which were covered by columnar epithelium. In other areas, the growth pattern demonstrated florid fibroadenomatoid architecture. The remaining viable tumour (~80% viable) was composed mostly of epithelial elements, with abundant primitive tubular and glomeruloid structures in around 75% of the viable tumour, with stromal (20%) and blastemal elements (5%) also present. There were no nephrogenic rests and no anaplasia.

The tumour of infant PD37585 was driven by biallelic inactivation of the *REST* tumour suppressor gene, and no *FOXR2* rearrangement was found. On histology, the tumour was classified as non-anaplastic Wilms tumour, composed of 60% blastema and 40% epithelium. There was a single perilobar nephrogenic rest.

The Wilms tumours of two school age children (PD52209 and PD52230) were composed purely of blastema.

**Description of the role of morphology in our case selection.**

We initially found the *FOXR2* rearrangement in one Wilms tumour of a newborn (PD49348). We then sequenced a second infant Wilms tumour (PD54846) and found that it also had a *FOXR2* rearrangement. We compared their microscopic descriptions, written independently by different pathologists in different countries, and noted significant similarities, with both tumours being described

as ‘fibro-adenomatoid’. The morphology of the tumours of the first two patients with *FOXR2*-rearrangement (PD49348 and PD54846) was thus noted incidentally.

A consultant pathologist who was involved in the study and aware of the association of morphology with this genetic change identified a further infant case with similar morphology (PD63760) and suggested that we sequence in our study. On sequencing it was found to have the *FOXR2*-rearrangement.

**Note on the laser capture microdissection of the tumours.**

The tumour of PD54846 (a *FOXR2*-mutant infant case) was microdissected, with each microdissection whole genome sequenced. The morphology of each microdissection has been reviewed by a consultant pathologist. Images are provided in Extended Data Figure 5 and a description of the tumour components (epithelia, stroma, or blastema) in each cut is provided in Supplementary Table 14. Please note that the morphology of laser capture is not to the same standards as clinical morphology due to constraints on tissue preparation: it has been frozen, thawed, and fixed in paxgene, and thicker sections are taken to maximise the DNA yield from each cut[1].

The spatial relationship between microdissections is shown in Extended Figure 5. Interestingly, there is no obvious correlation between the histology of the tissue making up the cut and the position on the phylogeny or the branch length. It appears that cells that are near each other in space tend to be related phylogenetically even if they have different morphology. This implies that a common ancestor existed in a spatially confined part of the tumour that was able to give rise to cells with each of the three histological appearances. If this is true, what matters in order to capture the true phylogeny of the tumour and to maximise resolution is to sample widely spatially, rather than sampling cells with particular histological appearances.

**Note on the comparison of mutation burden with morphology**

The tumours of the older children (PD52209 and PD52230) were composed of pure blastema. It is possible that this morphological appearance contributes to the difference in mutation burden between these tumours and that of the infant cases. Two pieces of evidence argue against this: first, the fact that there is no difference between the mutation burdens of blastema, epithelium, and stroma from PD54846; second the fact that PD52209 and PD52230 have mutation burdens that are typical for Wilms tumours (see Fig 1a), when those tumours have a range of histologies.

The four tumours with a strong discrepancy in mutation burden as derived by bulk whole genome sequencing and nanoseq were all those of infants. Three of these had the *FOXR2* rearrangement, and one had the embryonic *REST* mutation. The three with *FOXR2* rearrangement shared a the ‘fibro-adenomatoid’ morphological pattern described above, which the *REST*-mutant tumour did not have.

Although there is a correlation between the morphological pattern of the *FOXR2*-mutant infant cases with the discrepancy between bulk and duplex-derived mutation burdens, the fact that the *REST*-mutant infant tumour had different morphology but also had the discrepancy suggests that the morphology is not driving the discrepancy, although we do not have enough cases with different morphology to be conclusive.

**Note on the immunohistochemistry of the *FOXR2*-mutant tumours.**

The three infant Wilms tumours with *FOXR2* rearrangements noted by whole genome sequencing were tested by immunistochemistry for expression of the FOXR2 protein and the MYCN protein, which it is thought to stabilise[2]. Immunohistochemistry for FOXR2 was challenging. It was possible to show clear overexpression of FOXR2 and MYCN in two of the tumours (PD49348 and PD54846) (Supplementary Figure 1). For the third *FOXR2*-mutant infant Wilms tumour (PD63760), it was not possible to show FOXR2 overexpression by immunohistochemistry. This may be because it had been fixed in formalin for two months prior to staining. MYCN overexpression, however, was apparent. We note, incidentally, that in all three cases MYCN overexpression appears to be confined to the epithelial component of the tumour.

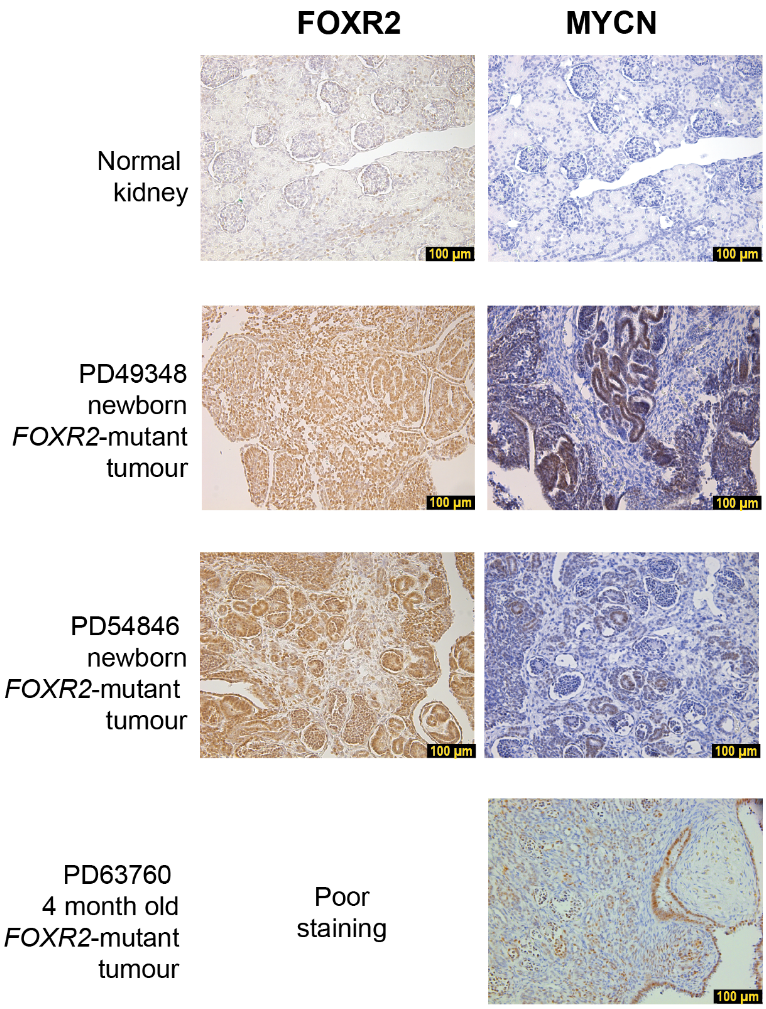


**Supplementary Figure 11. Immunohistochemistry for FOXR2 and MYCN.**

**Note on overall mutation burden within the tumour.**

Under a simple model, we can estimate the total number of substitutions within all cells of a tumour. Here, we estimate the number of mutations in the tumour of PD49348, an infant with a *FOXR2* rearrangement, using high-purity bulk whole genome sequencing, duplex sequencing, and organoid sequencing data. This analysis relies on four key pieces of information:

- The estimated number of cells in the tumour.
- The number of mutations found by bulk whole genome sequencing.
- The per-tumour cell mutation burden, as found by duplex sequencing.
- The number of clonal mutations, given by phylogenies from single cell derived organoid sequencing.

Phylogenetic analysis shows that the tumour arose from a single most recent common ancestor (MRCA) cell. We know that that the descendants of the most recent common ancestor cell grew to form a sizeable mass. DNA extraction quantification provides a minimum estimate of the tumour cell count. Analysis of allele fractions and bulk RNA sequencing indicates that most of the samples were predominantly tumour cells (Methods, Extended Data Fig. 2). Thus, we have an estimate of the tumour cell number for each biopsy.

For the tumour of PD49348, 40-50ug of DNA were extracted from each of four bulk tissue samples. Assuming 6.6pg of DNA per human cell, this equates to a minimum of approximately 6 million cell per bulk-sequenced sample. As there were four samples, we know that there were at least 24 million cells in the tumour, and likely many more, as only a small proportion of the tumour was used for sequencing. 25 generations of perfect doubling cell divisions are required to make 24 million cells (log2(24 million) = 24.5). We know from duplex sequencing that the mutation burden per cell was approximately 150 mutations per cell (using samples PD49348m, PD49348o, and PD49348p, which had the highest tumour cell fractions). Of these 150 mutations, 15 were truncal, as shown by the single cell derived organoid phylogenies. 135 (150 minus 15) mutations, therefore, were acquired per cell over a minimum of 25 doubling divisions. This equates to 5.4 mutations per doubling cell division, assuming a constant mutation rate. The total number of mutations in the tumour, therefore, is 5.4 mutations times by the total number of cell divisions that have occurred. The number of cell divisions that have occurred is equivalent to the number of edges in the rooted phylogeny. An edge leads to every internal node in the tree, save for the top node (since we are ignoring truncal mutations), and to every cell in the final generation (leaves, in phylogenetic terms). The number of internal nodes in a phylogeny is equal to the number of leaves minus one. Thus:

Edges = Leaves + internal nodes -1

Edges = leaves + (leaves -1) – 1

Edges = 2*(leaves -1)

Where

Edges == total number of cell divisions that have occurred

Leaves == total number of cells in the tumour at the time of sampling

The number of leaves is equal to the two to the power of the number of generations of doubling cell divisions (n).

Edges = 2*((2^n)-1)

Edges = 2^(n+1) - 2

If we assume 25 generations of doubling cell divisions:

Total divisions= (2^26)−2 = 67,108,862 divisions.

Therefore:

Total mutations = 67,108,862 divisions * 5.4 mutations/cell division

= 362,387,855 mutations.

If we assume 24 generations of doubling cell divisions:

Total divisions= (2^25)−1 = 33,554,430 divisions.

Therefore:

Total mutations = 33,554,431 divisions * 5.4 mutations/cell division

= 181,193,922 mutations.

This is likely an underestimate, as there were probably many more cells than we observed in the tumour.

We can check if this estimate aligns with our bulk whole genome sequencing result. Only mutations with a variant allele fraction (VAF) of greater than 0.1, approximately, are detectable by standard bulk whole genome sequencing, even in a high purity tumour such as ours. We can estimate the number of generations of cell divisions that are detectable by bulk sequencing. Truncal mutations, that are present in every tumour cell, have a VAF of 0.5 (as cells are diploid). Mutations acquired in the first generation after the trunk will have VAFs of approximately 0.25, and the second generation will have VAFs of

approximately 0.125, and so on. Only the first two generations, therefore, can be detected by standard sequencing, if clone sizes remain balanced. Using this to calculate the number of mutations that can be detected by bulk whole genome sequencing:

- Truncal mutations: 1 clone * 15 mutations = 15 mutations
- First post truncal generation: 2 clones * 5.4 mutations= 11 mutations
- Second post truncal mutations: 4 clones * 5.4 mutations. = 22 mutations

The total number of mutations, therefore, is 15 + 11 + 22 = 48 mutations.

We found 30-40 mutations by bulk sequencing each sample, which is not dissimilar to our estimate of 48. The fact that the observed bulk burden is lower than our estimate of 48 suggests that the mutation burden per symmetrical cell division may be lower. We can estimate the number of mutations per cell division based on the whole genome sequencing information, and apply it back to our model above.

We observe 36 mutations (median) in our bulk whole genome sequenced samples.
15 of these mutations are truncal. 21 mutations, therefore, remain to be accounted for, over two generations of doubling cell divisions, and so between six clones (two from the first doubling cell division, four from the second). 21/6=3.5 mutations per cell division. We can now combine this with our duplex sequencing estimate of 150 mutations per cell. 135 subclonal mutations per cell, divided by 3.5 mutations per doubling cell division, result in 38.6 doubling divisions. This would result in 2^38 = 274,877,906,944 cells. If all these cells were extant, that would imply 3.5*(2^38) =962,072,674,304 mutations total within the tumour. If due to cell death, only 24 million of these cells remained, then the number of mutations in the tumour would be 3.5 * 33,554,431 = 117,440,508 mutations (24 doubling divisions) or 3.5 * 67,108,863 = 234,881,020 mutations (25 doubling divisions).

If there is variation in clone sizes, then the number of generations of mutations called by bulk whole genome sequencing may be higher. The 36 mutations may go, say, to the 5$^{th}$ generation after the trunk. This would imply a lower mutation rate per doubling cell division. The effects of clonal imbalances are bounded: the mutation rate is likely to be at least 1 mutation per doubling cell division, and we know that clonal imbalance cannot be too strong or all the single cell derived organoids would be likely to be picked from the same subclone, and so the trunk would appear longer.

We set out to test the effect of clonal imbalance through simulation. One plausible method of achieving variation in clonal proportions in the absence of subclonal driver mutations is through bottlenecks. We simulated exponential growth after 15 truncal mutations to reach a population size of 2^25 cells, with different numbers of mutations per doubling cell division and bottlenecks removing 90% of the cells occurring at different points in the tumour's evolution. Whole genome sequencing mutation calling and duplex sequencing mutation calling were simulated on the output data. The code for simulations is available at https://github.com/HLee-Six/infant_Wilms.

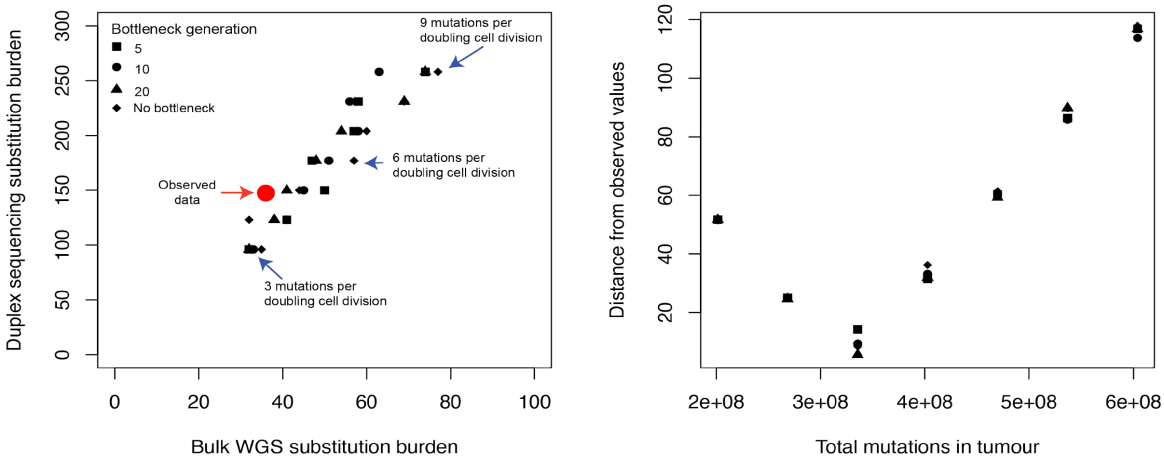


**Supplementary Figure 12. Simulations of the total mutation burden of an infant Wilms tumour.** Simulations were carried out as described in the text. **a**, the mutation burden from bulk whole genome sequencing (WGS) and duplex sequencing is shown for each simulation in black, while the observed result is shown in red. The timing of bottlenecks is shown by the plot symbol. **b**, the Euclidean distance of every simulation to the observed data (y axis) is shown against the total number of mutations in every cell in in the simulated tumour (x axis).

Simulation results indicate that bottlenecks have a fairly small effect and suggest a total number of mutations per doubling cell division of around 5. The total number of mutations per tumour in simulations that gave mutations burdens most similar to our data was 335,544,330. This is similar to the calculation value of 362,387,855 mutations. The lower value in the simulation is not surprising given than 5 mutations per generation were used in these simulations, compared to 5.4 mutations per generation in calculations.

We have a simple model of exponential growth with all cells within a generation dividing at the same time. We assume a constant mutation rate, and only apply a single bottleneck. This model is, of course, an oversimplification, and many other models of tumour growth may be applied. Altering the model, however, is unlikely to change the result of total numbers of mutations by orders of magnitude. Both by calculation and by simulation, the total number of mutations in the tumour of PD49348 exceeds 100 million, a far cry from the 36 mutations found by bulk whole genome sequencing.

## SUPPLEMENTARY REFERENCES.


1. Ellis, P. *et al.* Reliable detection of somatic mutations in solid tissues by laser-capture microdissection and low-input DNA sequencing. *Nat. Protoc.* 16, 841–871 (2021).
2. Schmitt-Hoffner, F. *et al.* FOXR2 Stabilizes MYCN Protein and Identifies Non–MYCN-Amplified Neuroblastoma Patients With Unfavorable Outcome. *J. Clin. Orthod.* **39**, 3217–3228 (2021).
