# Supplementary material for: High resolution clonal architecture of hypomutated Wilms tumours
Source: Nat Commun. 2025 May 29;16:4647. doi: 10.1038/s41467-025-59854-4 (PMC12122882; doi:10.1038/s41467-025-59854-4)
Supplement: Supplementary file 2 — Reporting Summary [file 41467_2025_59854_MOESM2_ESM.pdf]

nature portfolio

Corresponding author(s): Sam Behjati

Last updated by author(s): 18/03/2025

Reporting Summary

Nature Portfolio wishes to improve the reproducibility of the work that we publish. This form provides structure for consistency and transparency in reporting. For further information on Nature Portfolio policies, see our Editorial Policies and the Editorial Policy Checklist.

Statistics

For all statistical analyses, confirm that the following items are present in the figure legend, table legend, main text, or Methods section.

n/a	Confirmed	
☐	☒	The exact sample size (*n*) for each experimental group/condition, given as a discrete number and unit of measurement
☐	☒	A statement on whether measurements were taken from distinct samples or whether the same sample was measured repeatedly
☒	☐	The statistical test(s) used AND whether they are one- or two-sided<br>*Only common tests should be described solely by name; describe more complex techniques in the Methods section.*
☒	☐	A description of all covariates tested
☐	☒	A description of any assumptions or corrections, such as tests of normality and adjustment for multiple comparisons
☐	☒	A full description of the statistical parameters including central tendency (e.g. means) or other basic estimates (e.g. regression coefficient) AND variation (e.g. standard deviation) or associated estimates of uncertainty (e.g. confidence intervals)
☒	☐	For null hypothesis testing, the test statistic (e.g. *F*, *t*, *r*) with confidence intervals, effect sizes, degrees of freedom and *P* value noted<br>*Give P values as exact values whenever suitable.*
☒	☐	For Bayesian analysis, information on the choice of priors and Markov chain Monte Carlo settings
☒	☐	For hierarchical and complex designs, identification of the appropriate level for tests and full reporting of outcomes
☒	☐	Estimates of effect sizes (e.g. Cohen's *d*, Pearson's *r*), indicating how they were calculated

Our web collection on statistics for biologists contains articles on many of the points above.

Software and code

Policy information about availability of computer code

Data collection	NA
Data analysis	Published software was used as referenced. Publicly available code is referenced in the manuscript (please see Methods and Supplementary Note). Software versions used were: CaVEMan algorithm (version 1.15.1), vafCorrect (https://github.com/cancerit/vafCorrect, version 5.6.0), cgpPindel version 3.5.0.<br><br>Custom code is available on github at https://github.com/HLee-Six/infant_Wilms.

For manuscripts utilizing custom algorithms or software that are central to the research but not yet described in published literature, software must be made available to editors and reviewers. We strongly encourage code deposition in a community repository (e.g. GitHub). See the Nature Portfolio guidelines for submitting code & software for further information.

Data

Policy information about availability of data

All manuscripts must include a data availability statement. This statement should provide the following information, where applicable:

- Accession codes, unique identifiers, or web links for publicly available datasets
- A description of any restrictions on data availability
- For clinical datasets or third party data, please ensure that the statement adheres to our policy

Sequencing calls described in this article are provided in the supplementary information. Raw DNA data generated in this study have been deposited in the European Genome-Phenome Archive (EGA) under accession code EGAD00001009812. Raw RNA data generated in this study have been deposited in the European Genome-Phenome Archive (EGA) under accession code EGAD00001009813. Access to these datasets is restricted due to data privacy laws although access may be granted following an application to the Data Access Committee.

Research involving human participants, their data, or biological material

Policy information about studies with human participants or human data. See also policy information about sex, gender (identity/presentation), and sexual orientation and race, ethnicity and racism.

Reporting on sex and gender	Not reported.
Reporting on race, ethnicity, or other socially relevant groupings	Not reported.
Population characteristics	Not reported.
Recruitment	Samples were obtained from either the Princess Máxima Center biobank in the Netherlands, or from the SIOP2001 or IMPORT, now known as UMBRELLA, studies. Informed consent to participate, including use of tissue samples for research, was obtained from all the participants in both studies.
Ethics oversight	The Máxima biobank protocol was approved by the Medical Ethics Committee of the Erasmus Medical Center in Rotterdam, The Netherlands, under reference number MEC-2016-739. Approval for use of the subject's data within the context of this study was granted by the Máxima biobank and data access committee (https://research.prinsesmaximacentrum.nl/en/core-facilities/biobank-clinical-research-committee), biobank request number PMCLAB2018-006. The IMPORT study was approved by the NHS National Research Ethics Service Committee London - London Bridge (12/LO/0101). The SIOP2001 study was approved by East Midlands – Derby Research Ethics Committee (National Research Ethics Service (NRES) in the UK) (reference approval number MREC/01/4/086, from 17.01.2002).

Note that full information on the approval of the study protocol must also be provided in the manuscript.

Field-specific reporting

Please select the one below that is the best fit for your research. If you are not sure, read the appropriate sections before making your selection.

☒ Life sciences ☐ Behavioural & social sciences ☐ Ecological, evolutionary & environmental sciences

For a reference copy of the document with all sections, see nature.com/documents/nr-reporting-summary-flat.pdf

Life sciences study design

All studies must disclose on these points even when the disclosure is negative.

Sample size	Sample size for the cohort of infantile Wilms tumours was determined by the low frequency of the disease and the cost of high resolution investigation.
Data exclusions	One infantile case for which there was no suitable tissue for phylogenetic reconstruction and in which no driver mutation could be found was not described.
Replication	The key findings were ascertained through multiple different methods (organoid and laser capture microdissection, whole genome and duplex sequencing).
Randomization	No randomisation was involved.
Blinding	There was no bliding.

Reporting for specific materials, systems and methods

We require information from authors about some types of materials, experimental systems and methods used in many studies. Here, indicate whether each material, system or method listed is relevant to your study. If you are not sure if a list item applies to your research, read the appropriate section before selecting a response.

Materials & experimental systems

n/a	Involved in the study	
☐	☒	Antibodies
☒	☐	Eukaryotic cell lines
☒	☐	Palaeontology and archaeology
☒	☐	Animals and other organisms
☐	☒	Clinical data
☒	☐	Dual use research of concern
☒	☐	Plants

Methods

n/a	Involved in the study	
☒	☐	ChIP-seq
☒	☐	Flow cytometry
☒	☐	MRI-based neuroimaging

Antibodies

Antibodies used	The following primary antibodies were used: anti-FOXR2 (Abcam, ab244513, 1:1,000) and anti-c-Myc/N-Myc (D3N8F) (Cell Signaling Technology, #13987, 1:200).
Validation	No direct antibody validation has been carried out. The expression of FOXR2 is consistent with the RNA-seq data.

Clinical data

Policy information about clinical studies

All manuscripts should comply with the ICMJE guidelines for publication of clinical research and a completed CONSORT checklist must be included with all submissions.

Clinical trial registration	The patients were not part of a clinical trial.
Study protocol	Samples were obtained from either the Princess Máxima Center biobank in the Netherlands, or from the SIOP2001 or IMPORT, now known as UMBRELLA, studies.
Data collection	Approval for use of the subject's data within the context of this study was granted by the Máxima biobank and data access committee (https://research.prinsesmaximacentrum.nl/en/core-facilities/biobank-clinical-research-committee), biobank request number PMCLAB2018-006. The IMPORT study was approved by the NHS National Research Ethics Service Committee London - London Bridge (12/LO/0101). The SIOP2001 study was approved by East Midlands – Derby Research Ethics Committee (National Research Ethics Service (NRES) in the UK) (reference approval number MREC/01/4/086, from 17.01.2002). Informed consent to participate, including use of tissue samples for research, was obtained from all the participants in both studies.
Outcomes	Not applicable.

Plants

Seed stocks	NA.
Novel plant genotypes	NA
Authentication	NA
